# Dr. Roberton, on Hydrocephalus Internus

**Published:** 1810-09

**Authors:** John Roberton

**Affiliations:** Princes Street


### *To the Editors of the Medical and Phyſical Journal.*

Gentlemen,

I Request you will have the goodness to insert the following Answer to certain Remarks which appeared in No. 131 of the Medical and Physical Journal, upon a Paper of mine lately lately inserted, on the subject of Hydrocephalus Internus. The Gentleman, I am sorry to say, with perhaps great submission to his judgment on other subjects, has totally misunderstood every thing respecting the leading principle which I have advanced in the Paper alluded to.

It will only be necessary for me to state to the gentleman, what I have first of all asserted, in my Treatise on Medical Police, &c. lately published, and what I have since published in addition to that in Number 129, alluded to by the gentleman, viz. that I conceive the disease, known by the term hydrocephalus internus, to be an inflammatory affection of the whole system, and the brain and its membranes as a part of the whole; and that effusion of water into the ventricles of the brain is only a *consequence* of these inflammatory symptoms having either been entirely neglected, or perhaps, what is worse, ill treated. On this subject I suggested, what I can scarcely believe possible to be misunderstood by one of even very common abilities, or misconceived but by total neglect, that such a state of the system was perhaps most easily ascertained *by attention to the state of the pulse.* This, I say, I stated to be *the disease,* and *that, the method* by which it might almost to a certainty be ascertained, and if early attended to, removed. As concomitant symptoms, though not always present, I stated that the bowels were, *in general,* very torpid even from the first, and the stools black, or at least brown or green; (this the gentleman has imagined I stated to be the only cause and principal symptom of this widely spreading disease; but attention to the works above alluded to will I think convince him, if he will be convinced, that I meant no such thing) and that later in the disease great torpidity of the whole system was also very common, though not invariably present; and existed in some even to such a pitch that no artificial means could rouse it into any ordinary degree of action, nor could blisters produce any effect on the integuments of the head.

In respect to the prognosis of this disease, I stated, I think very plainly, that our chances of affording relief existed only *previous to effusion into the ventricles of the brain;* that this state existed always an indefinite number of days, in some even the length of one, two, or three weeks, and that when effusion had once taken place, the disease was in its last stage, and then *always irremediable.*

I therefore agree with the gentleman in conceiving the disease after effusion (the only state previous to my publi-

 cation cation on the subject in which it ever has been described by writers, or deemed to exist by medical practitioners, and from which it has even derived its name) to be irremediable; and in order to prevent that occurrence, our attention, if we expect to be successful, must be directed to the early, consequently to the inflammatory, stage of the disease, when in almost every case it is easily manageable. At this important crisis, I repeat it, our sole object is to reduce inflammatory action. For this purpose, as in every other disease, the bowels, whether torpid or otherwise, must be freely opened, a cap blister put all over the upper part of the head,* and, should the inflammatory action be obstinate, general bleeding, the application of leeches to the temples, and the laying open the jugular vein or the temporal artery is absolutely necessary.

Although I am accused by the gentleman of forming as vague theories as any one else, I can assure him that unless theories can lead to a rational train of practice I uniformly abandon them. None are less apt than I am to indulge in visionary speculations, where either the improvement of my profession will suffer by them, or the safety and comfort of the distressed are in any way likely to be lessened. I believe the only theoretical paragraph in my essay on this subject, in my work on Medical Police, is what I shall here subjoin, and it was not without extensive observation and considerable reflection that I ventured to advance it. It bears internal marks of probability.

"Its (hydrocephalus internus) attacking infants in preference to adults might be explained. We know that such is the particular state of the body in children, that the propelling and resisting powers have not acquired that degree of independence of each other, which they possess in after life, so that their diseases are totally different from those of adults; for, from this state of the body, any disease, wherever situated, will, either primarily or ultimately, affect the most delicately organized and tender parts in preference to any other;" thus I conceive it is, that the brain of children is so subject to this disease.

In

The cruelty of this practice the author of the paper in Number 131, declaims against most bitterly. I can assure the gentleman that I have scarcely ever heard any infant even cry from the pain occasioned by such a blister; but, on the contrary, have observed them greatly relieved almost instantly. In short, the sole object of the author of the paper in Number 131 must amount to this;—rather allow the infant to die to a certainty than even occasion to it temporary inconvenience by a blister.

In respect then to my theory of the disease, and the practice founded upon it, I believe the gentleman will find that, by steadily examining the nature of the one, and the result of the other, he will be agreeably surprised to find simplicity take the place of former confusion, and success of disappointment. I wish the gentleman to understand that it is upon previous rational theory that I conceive it to be possible to establish scientific practice. It is not by wild hypothetical flights, which some men call reasoning, that we are to expect any immediate practical advantage. Hypotheses are less definite than theory, and are, in all instances, the first operation of our minds in the investigation of difficulties in science. Hypothesis, therefore, ought to exist no longer than we can possibly transform it into theory. On this we must build our practice, and this practice will be successful or otherwise in proportion as we have theorised.

I have no hesitation in asserting, that, previous to the very little I have written on this subject in the works above alluded to, no theory of any kind existed respecting this disease. What was said about it amounted, at least to the thinking part of the community, to very little. The disease was never till then considered in its first stage, *when effusion had not yet taken place*, and when *alone* it was remediable. We do not hesitate to blame any one for allowing every variety of the most fatal complaints, such as fevers, &c. &c. to go on unchecked till the last stages, and then most feelingly pronounce the disease to be incurable, which in almost every instance could be easily removed by attention to their early stages. Though, however, we do not approve of this, we silently allow the most precious moments to elapse in the disease in question, and when it is really incurable we take to ourselves the *merit* of saying so. Nevertheless, not only for this, but, as in No. 131 of this Journal, for my making an attempt to improve the reasoning and practice, in a disease acknowledged by every one, and even by the gentleman himself, to be incurable, I am found fault with.

With regard to the opinion either of the public at large, or any individual of it, in respect to any point in my profession, I pay them equal deference, and hold them in the very highest respect when I conceive it to be right. But when the whole or any individual of it, however high he may stand in the estimation of the world, advances opinions which seem *to me* erroneous, I never hesitate to state my objections, and, if possible, propose something which I

think less objectionable. Names I regard not in such matters, nor shall they ever deter me from the investigation of any point. Neither do I wish or expect that they shall either influence or deter others from making the freest and most liberal remarks on any opinions I may have or shall hereafter advance. Names are the stumbling blocks of all improvement, and their influence is always directly in proportion to our want of real knowledge.

I shall then be happy to hear the unbiased opinions of scientific men on any or every opinion I may make public, and, if objected to, I shall either openly confess myself in the fault, or, in the same open manner, I pledge myself to defend the doctrines I have espoused or may yet espouse. In the mean time, when your Correspondent has attentively consulted the works I have named, I shall be glad to hear his further observations.

Another paper appeared in your Journal some time previous to the one I have alluded to, but as I have not the Journal at hand at present, I do not recollect either the author's name or in what number it was inserted. The gentlemen seem acquainted with each other; and as the objections there stated were of nearly the same nature as those I have now alluded to, the same answer will do for both.

I am, &c.

*Princes Street, July* 23, 1810. JOHN ROBERTON.

